# The Inhibition of Subchondral Bone Lesions Significantly Reversed the Weight-Bearing Deficit and the Overexpression of CGRP in DRG Neurons, GFAP and Iba-1 in the Spinal Dorsal Horn in the Monosodium Iodoacetate Induced Model of Osteoarthritis Pain

**DOI:** 10.1371/journal.pone.0077824

**Published:** 2013-10-30

**Authors:** Degang Yu, Fengxiang Liu, Ming Liu, Xin Zhao, Xiaoqing Wang, Yang Li, Yuanqing Mao, Zhenan Zhu

**Affiliations:** Department of Orthopaedics, Shanghai Ninth People’s Hospital, Shanghai Jiao Tong University, School of Medicine, Shanghai, People’s Republic of China; University of Arizona, United States of America

## Abstract

**Background:**

Chronic pain is the most prominent and disabling symptom of osteoarthritis (OA). Clinical data suggest that subchondral bone lesions contribute to the occurrence of joint pain. The present study investigated the effect of the inhibition of subchondral bone lesions on joint pain.

**Methods:**

Osteoarthritic pain was induced by an injection of monosodium iodoacetate (MIA) into the rat knee joint. Zoledronic acid (ZOL), a third generation of bisphosphonate, was used to inhibit subchondral bone lesions. Joint histomorphology was evaluated using X-ray micro computed tomography scanning and hematoxylin-eosin staining. The activity of osteoclast in subchondral bone was evaluated using tartrate-resistant acid phosphatase staining. Joint pain was evaluated using weight-bearing asymmetry, the expression of calcitonin gene-related peptide (CGRP) in the dorsal root ganglion (DRG), and spinal glial activation status using glial fibrillary acidic protein (GFAP) and ionized calcium binding adaptor molecule-1 (Iba-1) immunofluorescence. Afferent neurons in the DRGs that innervated the joints were identified using retrograde fluorogold labeling.

**Results:**

MIA injections induced significant histomorphological alterations and joint pain. The inhibition of subchondral bone lesions by ZOL significantly reduced the MIA-induced weight-bearing deficit and overexpression of CGRP in DRG neurons, GFAP and Iba-1 in the spinal dorsal horn at 3 and 6 weeks after MIA injection; however, joint swelling and synovial reaction were unaffected.

**Conclusions:**

The inhibition of subchondral bone lesions alleviated joint pain. Subchondral bone lesions should be a key target in the management of osteoarthritic joint pain.

## Introduction

Osteoarthritis (OA) is a leading cause of disability in elderly people, and it is characterized by a progressive loss of articular cartilage, subchondral bone lesions and synovitis [1]. Chronic pain is the most prominent and disabling symptom of OA [2], but the causes of joint pain are not clear [3]. The search for effective agents that relieve joint pain has been challenging.

Articular cartilage and synovial inflammation have been the primary targets of pharmaceutical therapies for decades to attenuate joint degeneration and alleviate joint pain [4], [5], [6]. However, the role of subchondral bone has been largely ignored. Accumulating clinical evidence suggests that abnormal subchondral bones are a primary source of joint pain in OA. Bone attrition, which is defined as a flattening or depression of the subchondral bone, is strongly associated with the presence of pain in knee OA that is detected on X-ray [7] and magnetic resonance images (MRIs) [8]. The percentage of denuded subchondral bone plate is associated with moderate to severe knee pain using specialized MRI techniques [9]. Bone marrow lesions (BMLs) in the subchondral trabecular bone, which are ill-defined areas of high signal intensity on T2-weighted, fat-suppressed MRIs, are positively associated with knee pain in OA [10], [11]. Furthermore, a clinically dramatic attenuation of pain is often achieved with high tibial osteotomy, in which high levels of stress stimulation on localized abnormal subchondral bones are relieved by a realignment of the bones and a rebalancing of load [12]. Immediate joint pain relief is usually accomplished by total or unicompartmental knee arthroplasty, in which the majority of the affected subchondral plate is excised [13]. These clinical findings suggest that subchondral bone lesions contribute to the occurrence of joint pain in OA.

The most commonly used OA model for the study of joint pain is the injection of monosodium iodoacetate (MIA) into the knee of rats, which induces cartilage damage, aggressive subchondral bone lesions, inflammation and joint pain, and mimics the pathological changes and pain of osteoarthritis in humans [14], [15], [16], [17], [18]. This model produces alterations in the expression of neurochemical markers in the dorsal root ganglions (DRGs) and spinal cord dorsal horns that innervate the affected knee joint [19], [20], [21], [22], [23]. Zoledronic acid (ZOL), a third-generation bisphosphonate, inhibits osteoclastic bone absorption, and this agent exerts a protective effect on the subchondral bone in OA [24].

The present study investigated the inhibition of subchondral bone lesions on osteoarthritic joint pain. Pain behavior, knee joint histomorphology, and the expression of pain-related neurochemical indexes in the DRGs and spinal cord dorsal horns that innervate the osteoarthritic joints were evaluated in MIA-injected rats in the presence and absence of ZOL.

## Methods

### Induction of OA

Adult male Sprague Dawley rats (12 weeks old) from Sino-British Sippr/BK Lab Animal Ltd. (Shanghai, China) were used in the present study. The animals were group housed under a 12-hour light/dark cycle with food and water ad libitum. The Animal Care and Experiment Committee of the Shanghai Jiao Tong University School of Medicine approved all experimental procedures.

The rats were anesthetized with 10% chloral hydrate in phosphate-buffered saline (PBS, 0.01 M) after 1 week of acclimatization. The MIA model was induced as described previously. Briefly, 3 mg MIA (Sigma, I2512) in a total volume of 25 ul PBS was injected intra-articularly through the patellar ligament of the right knee using a 30-gauge needle and a Hamilton microsyringe. The sham joint was injected with the same volume of PBS.

### Experimental Design

A total of 48 animals were used in the present study. ZOL treatment was initiated immediately post-MIA injection. ZOL (Aclasta®, Novartis Pharma Stein AG) was administered subcutaneously twice per week at 100 µg/kg body weight (MIA/ZOL). The same volume of normal saline was injected in the Sham (Sham/NS) and MIA control animals (MIA/NS). The animals (n = 8 per group) were sacrificed at 3 and 6 weeks following OA induction. The dose of ZOL was based on previous studies [24], [25].

### Joint Swelling

Knee diameters were measured to determine the extent of joint tissue swelling as an index of inflammation following intra-articular MIA injections. The mediolateral diameters of the knee joint were measured with a digital micrometer (293-240, Mitutoyo, Japan) 1 day before and 3 and 6 weeks after MIA or PBS injection.

### Pain Behavioral Testing

Pain behavior was measured as a weight-bearing asymmetry between the OA-induced (ipsilateral) and contralateral limb using an incapacitance meter (IITC Life Sciences, Woodland Hills, CA, USA) as described previously [16]. Briefly, rats were placed in an angulated Perspex container, and each hind paw rested on a separate transducer pad. The force that was exerted by each hind limb was averaged over a 5-second period. Each data point is the mean of three 5-second readings. Changes in weight-bearing asymmetry were evaluated one day before and 3 and 6 weeks after MIA or PBS injection. The percentage of the weight placed on the right hind limb was determined using the formula below [16].
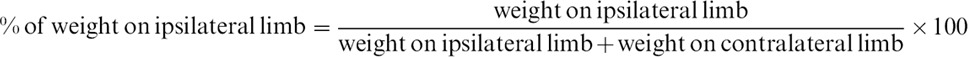



### Retrograde Neuronal Labeling

Afferent neurons in the DRGs that innervate the joint were identified using retrograde fluorogold (FG) labeling. FG (5 µl of 2%, Sigma, 39286) in 0.1 M PBS was injected intra-articularly into the right knee of an anesthetized rat using a 30-gauge needle and a Hamilton microsyringe one week prior to sacrifice. The FG dose was based on previous literature [20].

### Tissue Preparation

Anesthetized rats (n = 8 per group) were transcardially perfused with 250 ml heparinized saline (10 IU/ml) followed by 300 ml ice-cold, fresh 4% paraformaldehyde (PFA) in 0.1 M PBS. The lumbar L4 DRGs and spinal cord at the level of the lumbar enlargement were harvested, fixed in 4% PFA for 3 hours, and cryoprotected in 30% sucrose in 0.1 M PBS overnight at 4°C. The tissues were embedded in Tissue-Tek OCT compound (SAKURA Finetechnical, Tokyo, Japan) and stored at −80°C until sectioning. The entire knee joint was dissected for histomorphology evaluation.

### Micro-CT Imaging

**Figure 1:**
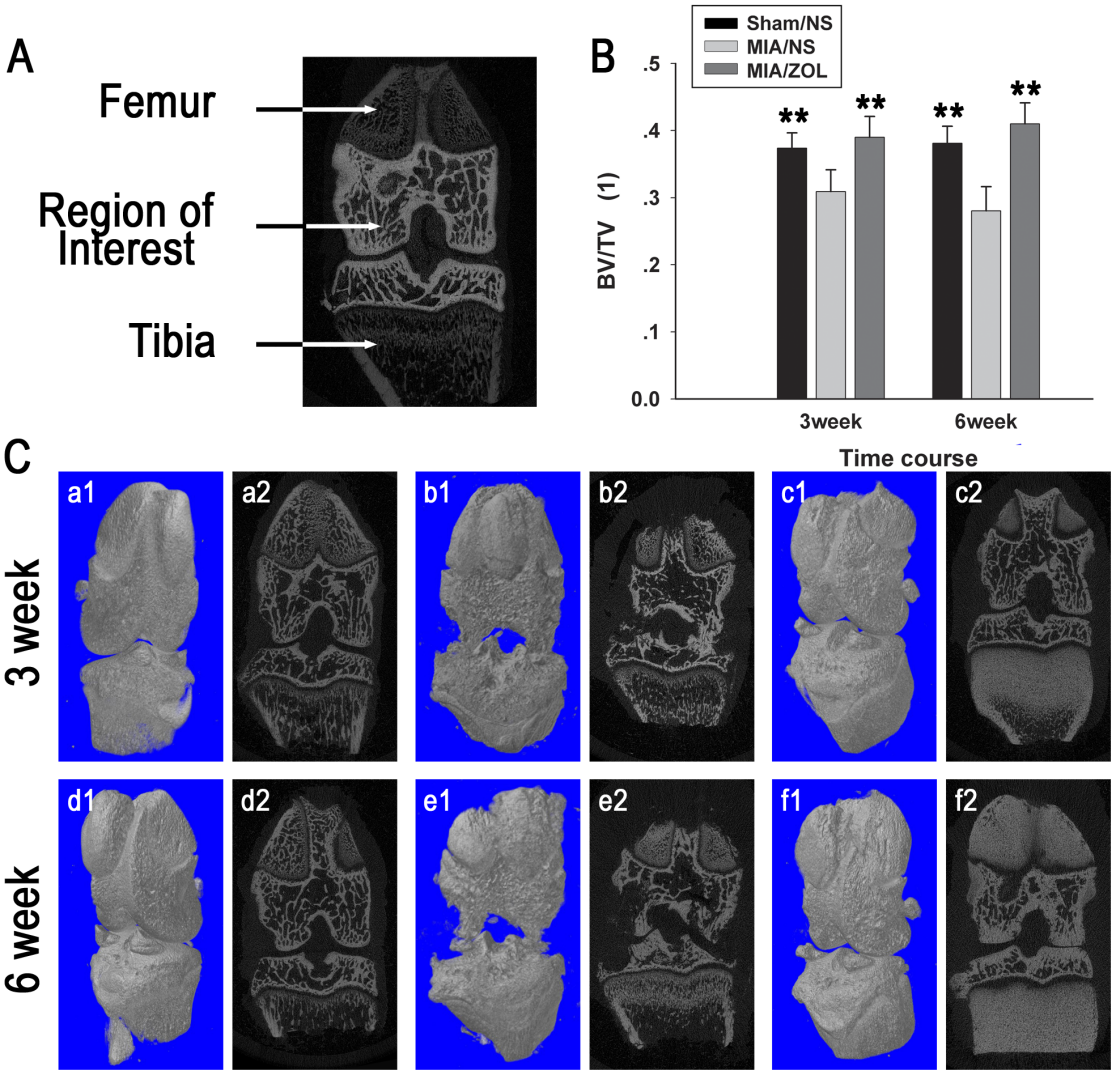
Alterations in subchondral bone architecture on micro-CT scans. A, Regions between the distal growth plate of the femur and the proximate growth plate of the tibia were selected as regions of interest. B, Analysis of bone volume fraction (BV/TV). Asterisks (**) denote *P*<0.01 compared to the MIA/NS group. C, Representative micro-CT images of the knee. Sham/NS group: a1, a2, d1, d2; MIA/NS group: b1, b2, e1, e2; MIA/ZOL group: c1, c2, f1, f2.

Structural alterations in subchondral bone architecture were evaluated using X-ray micro computed tomography (micro-CT) scanning. The knee joints were fixed in 4% PFA for 48 hours, and the entire knee joints underwent micro-CT (µCT 80; SCANCO Medical AG) with an isotropic voxel resolution of 10 µm. The regions between the distal growth plate of the femur and the proximate growth plate of the tibia were selected as regions of interest (Fig. 1A) for bone volume fraction (BV/TV) analysis.

### Histological Analysis

The knee joints were prepared for subsequent evaluation of cartilage damage and synovial reaction using the OARSI recommendations for histological assessments of osteoarthritis in the rat [26]. Briefly, the knee joints were decalcified in 10% EDTA for 3 weeks. The joints were bisected along the collateral ligament in the frontal plane, and both sections were embedded in the same paraffin block. The samples were cut into 5-µm sections. Three sections from each knee at 200-mm steps were stained with hematoxylin-eosin (H&E) to evaluate articular cartilage damage and synovial reaction. Three sections from each knee underwent tartrate-resistant acid phosphatase (TRAP, Sigma, 387A) staining, according to the manufacturer’s instructions, to observe the activity of subchondral osteoclasts.

### Immunofluorescence Analysis

The expression of calcitonin gene-related peptide (CGRP), a pain-related neuropeptide, was analyzed in DRG neurons. The DRGs were sectioned into 12-µm sections, and every sixth section was used for the immunofluorescence analysis of CGRP and isolectin-B4 (IB4). Nissl staining was performed subsequently to quantify the total number of neurons in each section. IB4 labels small nonpeptidergic neurons, which are observed primarily in cutaneous afferent neurons but rarely in joints; this marker ensured that fluorogold injections were articulation specific [20]. The sections were incubated using the following conditions for CGRP and IB4 double-labeling. First, the sections were incubated with rabbit anti-CGRP (1∶2000, Sigma, C8198) overnight at 4°C, FITC-conjugated IB4 (0.5 µg/ml, Sigma, L2895) for 1 hour at room temperature (RT); next, the sections were incubated with anti-rabbit Alexa 488 (1∶500, Molecular Probes, A11034) for 1 h at RT; finally, the sections were labeled with a fluorescent Nissl stain (1∶50, Molecular Probes, N-21482) for 20 min at RT. The total numbers of FG-labeled neurons, the percentage of CGRP- and IB4-positive FG-labeled neurons, and the total number of Nissl-labeled neurons were determined. The results of each individual rat were averaged.

The spinal cords were sliced into 25-µm sections, and every tenth section underwent immunofluorescence analysis for glial fibrillary acidic protein (GFAP) and ionized calcium binding adaptor molecule-1 (Iba-1), which label activated astrocytes and microglia, respectively. The sections were prepared as follows. First, the sections were incubated with rabbit anti-GFAP (1∶500, Abcam, ab7779) overnight at 4°C or with rabbit anti-Iba-1 (1∶500, Wako, 019-19741) overnight at 4°C. Next, the sections were incubated with anti-rabbit Alexa 488/594 for 1 h at RT.

Quantification of microglia and astrocyte activation was based on previous methods [21], [22]. The number of Iba-1-positive microglia clearly swollen cell bodies per 10 mm^2^ and the mean intensity of GFAP immunofluorescence were calculated in the ipsi- and contralateral dorsal horns.

All images were acquired using a fluorescence microscope (Leica DM 4000B) with BioQuant OSTEO II software (BioQuant Image Analysis Corporation, Nashville, TN). The entire image of each DRG section was acquired at 200× magnification with automatic sequential imaging. The images were analyzed with Image-Pro Plus 6.0 (Media Cybernetics).

### Statistical Analysis

Blinded authors who were unaware of the treatments performed all knee diameters measurements, weight-bearing tests, micro-CT scans, histological assessments, and immunofluorescence analyses. The results are presented as the means ± standard deviation (SD). A comparison between groups was performed with a one-way analysis of variance using the LSD test. *P* values less than 0.05 were considered statistically significant.

## Results

### Knee Joint Swelling

**Figure 2:**
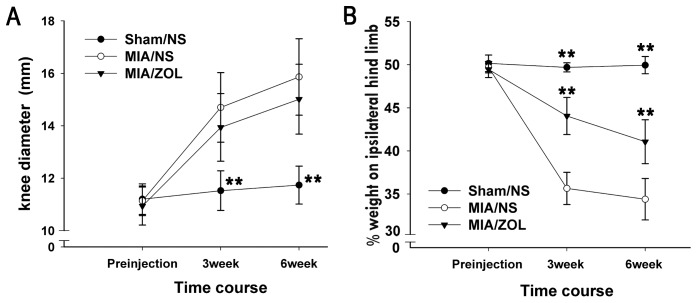
Changes in knee joint swelling and pain behavior. A, Knee joint swelling measured as knee diameter. B, Pain behavior determined as the percentage of weight borne on the ipsilateral hind limb. Asterisks (**) denote *P*<0.01 compared to the MIA/NS group.

MIA injection significantly increased knee joint diameter at both examined time points (Fig. 2A). The knee diameters of MIA/NS animals (14.7±1.3 mm at 3 weeks and 15.9±1.5 mm at 6 weeks) were not reduced in MIA/ZOL animals (13.9±1.3 mm at 3 weeks, *P*>0.05; 15.0±1.4 mm at 6 weeks, *P*>0.05). The knee diameters of Sham/NS animals were not significantly different between 3 and 6 weeks.

### Pain Behavior

Weight-bearing asymmetry between the ipsilateral and contralateral hind limbs was measured as an index of pain behavior. Intra-articular MIA markedly reduced hind limb weight bearing throughout the course of the experiment (Fig. 2B). ZOL treatment significantly reversed weight-bearing deficits compared to MIA/NS animals at 3 weeks (44.1±2.2% vs. 35.7±1.9%, respectively, *P*<0.01) and 6 weeks (41.1±2.6 vs. 34.4±2.4%, respectively, *P*<0.01).

### Alterations in Subchondral Bone Architecture

Micro-CT scans (Fig. 1) clearly demonstrated that intra-articular MIA induced significant alterations in the subchondral bone. Bone-cartilage interface collapse and subchondral bone loss occurred, and these alterations were aggravated 3 to 6 weeks post-MIA injection. ZOL treatment significantly inhibited these structural changes and preserved bone quantity. The BV/TV at 3 weeks was 0.39±0.031 in the MIA/ZOL group and 0.309±0.031 in MIA/NS animals, *P*<0.01. The BV/TV at 6 weeks was 0.41±0.031 in the MIA/ZOL group and 0.28±0.036 in MIA/NS animals, *P*<0.01. Marked osteosclerosis was observed at the tibial metaphysis in ZOL-treated animals, which was induced by ZOL inhibition of osteoclasts in the growth plate (Figs. 1c2 and 1f2).

### Histological Evaluation of the Knee Joint

**Figure 3:**
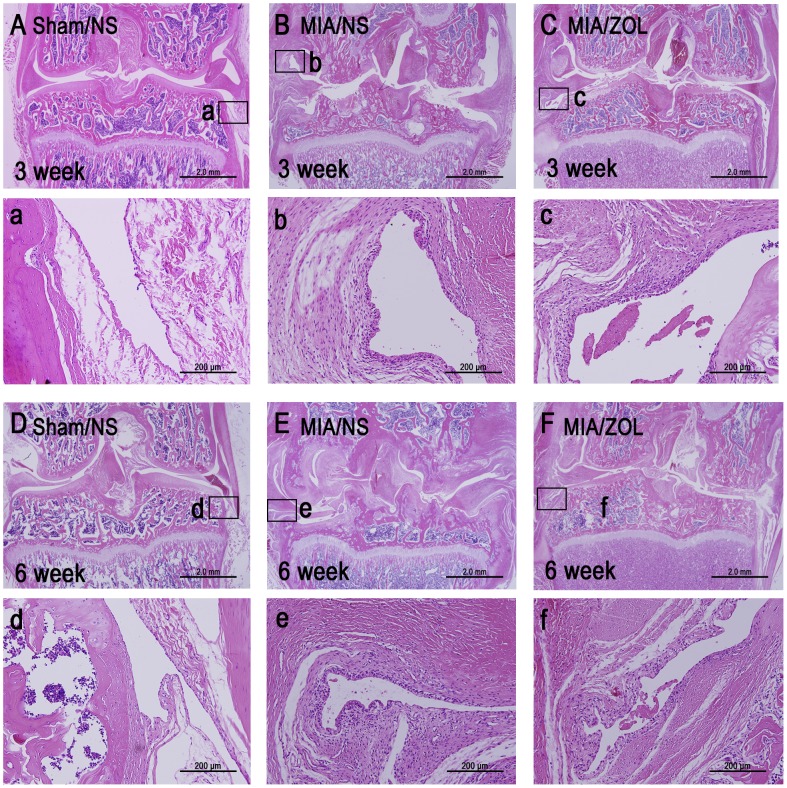
Representative images of histological alterations in the joint as indicated by H&E staining. One synovium site from every section is shown at 200× magnification.

MIA-injected joints exhibited significant histomorphological alterations, including articular cartilage loss, subchondral bone collapse and synovial reaction (Fig. 3). Full thickness and extensive cartilage loss occurred at 3 and 6 weeks. Subchondral bone collapse presented at 3 weeks, and this collapse was further aggravated 6 weeks after MIA injection. The Sham/NS knees displayed the normal 1–2 layers of synovial intima cells and no infiltration of inflammatory cells in the underlying loose connective tissue. MIA-injected knees exhibited significant synovial reaction with a 3–4 cell thick intimal hyperplasia and proliferation of subsynovial tissue but no obvious infiltration of inflammatory cells. ZOL treatment effectively preserved joint shape, inhibited subchondral bone loss, and delayed cartilage loss, but medial cartilage loss remained obvious at 6 weeks. However, no significant difference in the synovial reaction of MIA/ZOL animals compared to MIA/NS animals was observed. The synovial reaction scores for MIA/ZOL animals was 1.6±0.2 compared to 1.7±0.3 (*P*>0.05) for the MIA/NS animals at 3 weeks and 1.5±0.2 to 1.6±0.2, respectively, (*P*>0.05) at 6 weeks.

### Subchondral Osteoclast Activity

**Figure 4:**
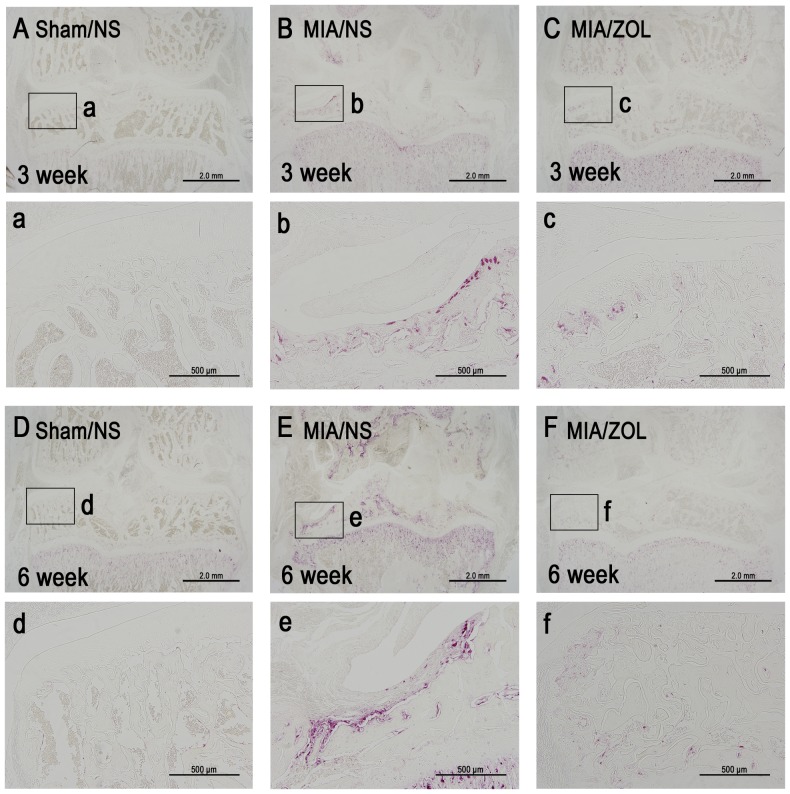
Representative images of subchondral osteoclast activity as indicated by TRAP staining. One site from every section is shown at 100× magnification.

Osteoclast activity was assessed using TRAP staining (Fig. 4). A large number of TRAP-positive multinucleated osteoclasts were present at the surface of the subchondral bone in the MIA/NS animals at 3 and 6 weeks. ZOL treatment significantly inhibited osteoclast activity in MIA/ZOL animals.

### Immunofluorescence Analysis of CGRP and IB4 Expression in L4 DRGs

**Figure 5:**
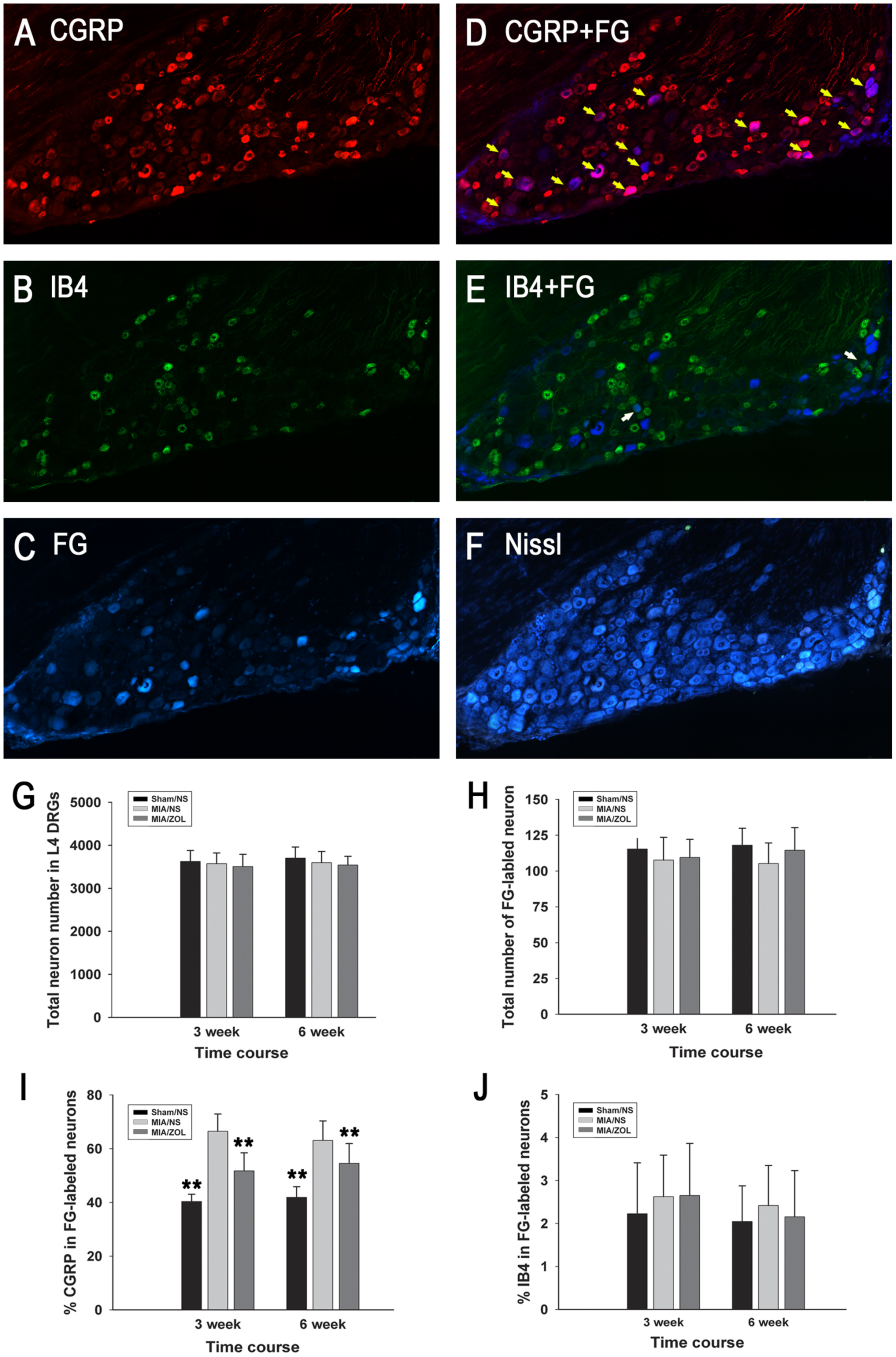
CGRP expression in DRG neurons. Representative images of the same DRG section labeled with CGRP (A), IB4 (B), FG (C), CGRP and FG (D), IB4 and FG (E), and stained with fluorescent Nissl (F). The yellow arrowheads in (D) indicate FG-labeled neurons that are CGRP-positive; the white arrowheads in (E) indicate FG-labeled neurons that are IB4-positive. The total number of neurons (G), the total number of FG-labeled neurons (H), the percentage of CGRP-positive neurons (I) and IB4-positive neurons (J) out of the total number of FG-labeled neurons. The results for each individual rat were averaged. Asterisks (**) denote *P*<0.01 compared to the MIA/NS group.

Alterations in the peripheral neurons of DRGs were evaluated using the expression of a pain-related neuropeptide, CGRP. Representative images are displayed in Fig. 5. The total neuron numbers were similar between groups (*P*>0.05) at 3 and 6 weeks. Similarly, the total numbers of FG-labeled neurons were not significantly different between groups (*P*>0.05). The percentage of CGRP-positive, FG-labeled neurons in MIA/NS animals (66.4±6.5% at 3 weeks; 63.1±7.3% at 6 weeks) was significantly greater than Sham/NS animals (40.3±2.7% at 3 weeks, *P*<0.01; 41.9±4% at 6 weeks, *P*<0.01). ZOL treatment significantly reduced the percentage of CGRP-positive, FG-labeled neurons compared to the MIA/NS group (51.7±6.7% at 3 weeks, *P*<0.01; 54.6±7.3% at 6 weeks, *P*<0.05). No differences in the expression of IB4-positive, FG-labeled neurons were observed between groups at 3 or 6 weeks.

### Immunofluorescence Analysis of GFAP and Iba-1 Expression in the Spinal Cord

**Figure 6:**
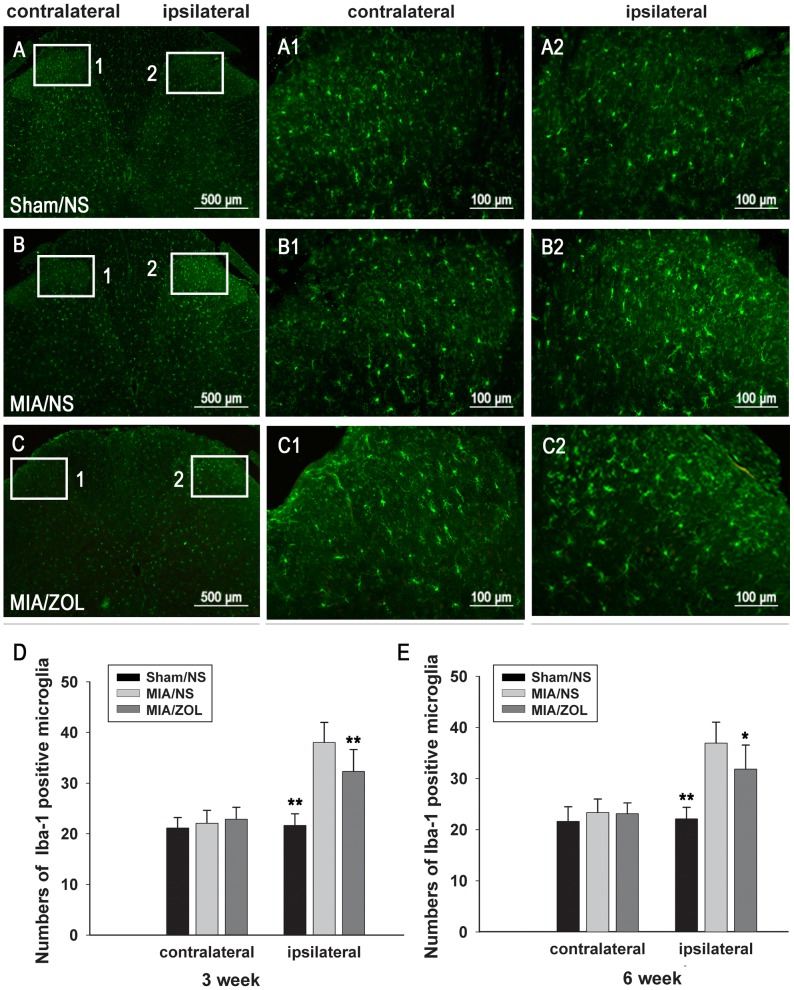
Changes in spinal microglia activation as evaluated by Iba-1 immunofluorescence. Representative images (A, B, C) are Iba-1 immunofluorescence 6 weeks after MIA injection. D and E, Analysis of the number of Iba-1-positive microglia; the results for each individual rat were averaged. (*) denotes *P*<0.05, and (**) denotes *P*<0.01 compared to the MIA/NS group.

**Figure 7:**
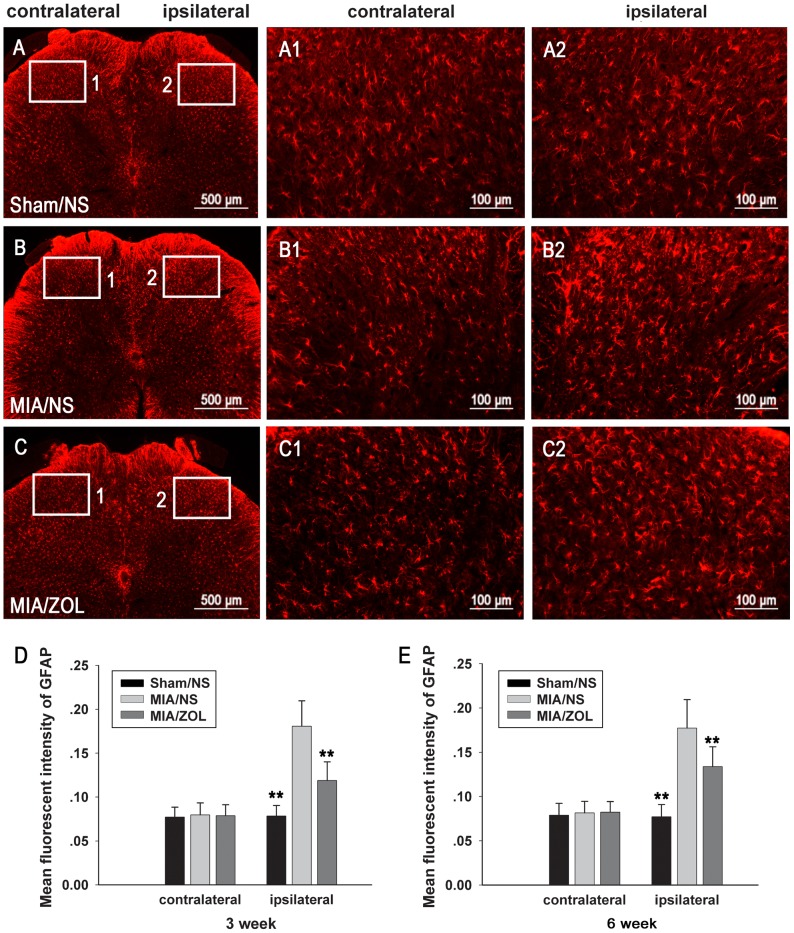
Changes in spinal astrocyte activation as evaluated by GFAP immunofluorescence. Representative images (A, B, C) are GFAP immunofluorescence 6 weeks after MIA injection. D and E, Analysis of the mean intensity of GFAP immunofluorescence; the results for each individual rat were averaged. Asterisks (**) denote *P*<0.01 compared to the MIA/NS group.

The spinal astrocytes and microglia were evaluated using immunofluorescence of Iba-1 and GFAP expression. Representative images of Iba-1 and GFAP immunofluorescence 6 weeks after MIA injection are presented in Figs. 6 and 7. The numbers of Iba-1-positive microglia in the ipsilateral spinal cord of MIA/NS animals were significantly increased (38.0±4 at 3 weeks, *P*<0.01; 36.9±4.1 at 6 weeks, *P*<0.01) compared to Sham/NS animals (21.6±2.3 at 3 weeks; 22.1±2.3 at 6 weeks). ZOL treatment significantly reduced the number of activated microglia at both time points (32.3±4.3 at 3 weeks, *P*<0.01; 31.8±4.7 at 6 weeks, *P*<0.05). Intra-articular MIA injections increased GFAP immunoreactivity. ZOL treatment significantly attenuated the MIA-induced increase in GFAP immunoreactivity. No significant differences in the expression of Iba-1 or GFAP were observed in the contralateral spinal cords between groups.

## Discussion

The well-established MIA model was employed to mimic clinically relevant OA pain in the present study. Intra-articular MIA injections induce a dose-dependent destruction of rat knees with a concomitant increase in joint pain [16], [24], [27], [28]. The present study demonstrated that an injection 3 mg MIA induced obvious joint pathology and joint pain, which is consistent with previous results. The preservation of the structural integrity of the subchondral bone using bone-modifying agents retards joint degeneration [25], [29], [30], [31], [32], [33], and the present study demonstrated a protective effect of ZOL on the subchondral bone via the inhibition of osteoclast activity. The present study further demonstrated that the inhibition of subchondral bone lesions by ZOL significantly attenuated the MIA-induced weight-bearing asymmetry, reduced the MIA-induced CGRP expression in DRG neurons, and suppressed the MIA-induced activation of microglia and astrocytes in the spinal cord. However, the synovial reaction and joint swelling were not affected.

The expression of pain-related neurochemical markers was investigated in the nervous system to better evaluate alterations in joint pain. Weight-bearing asymmetry between osteoarthritic and contralateral hind limbs is a measure of joint pain [18]. Our data demonstrated that intra-articular MIA injections produced a marked weight-bearing asymmetry, which is consistent with the results of previous studies [15], [16], [27]. ZOL treatment reversed this asymmetry. However, joint instability from joint structure destruction may also contribute to weight-bearing asymmetry. Joint pain has both peripheral and central (spinal and brain) components [2], [34], [35], [36]. Therefore, we also detected CGRP expression in DRG neurons and the activation of neurogliocytes in the spinal cord that innervated the affected joint. CGRP is a marker of sensory neurons, and it is involved in pain perception [37] and osteoarthritic joint pain [2], [20]. The present study demonstrated that ZOL treatment markedly attenuated the intra-articular MIA-induced increase in the number of CGRP-positive DRG neurons. The activation of neurogliocytes in spinal cord contributes to the establishment and maintenance of chronic joint pain [21], [22], [23]. The present study demonstrated that the activation of astrocytes and microglia in the spinal cord dorsal horn was greater in the MIA model, and these results are consistent with earlier studies [21], [22], [23]. We also demonstrated that ZOL treatment significantly attenuated spinal astrocyte and microglia activation in the OA model. Taken together with the behavioral data, our results indicate that ZOL treatment relieved MIA induced joint pain and attenuated the expression of pain-related neurochemical markers.

The origin of pain in OA is poorly understood [3]. Pathological changes in OA are characterized by cartilage loss, subchondral bone lesions, and synovial reaction [1]. Synovial reaction and subchondral bone lesions are positively associated with knee pain [38], [39]. Synovial reaction in OA, which involves synovial hyperplasia, subsynovial tissue proliferation and inflammatory cell infiltration, is a primary source of the inflammatory mediators that produce pain [5], [40]. However, knee swelling and synovial reaction were not different after ZOL intervention [24]. These results indicate that the ZOL-induced inhibition of OA structural changes, but not synovial reaction, alleviated joint pain. Adult articular cartilage is an aneural and avascular tissue, but these components are plentiful in subchondral bone [2], [41]. An overall analysis of our results suggests that the improvement in the subchondral bone reversed joint pain.

Several potential mechanisms may contribute to the pain relief following the inhibition of subchondral bone lesions. The subchondral bone is richly innervated [41], and the neurovasculature invades the osteochondral junction in osteoarthritis [42], [43], [44]. The mechanical properties of the subchondral bone are significantly reduced in OA [45], [46]. Therefore, subchondral nociceptors are prone to stimulation or injury by mechanical factors in OA. This increase in nociceptor sensitivity may contribute to weight-bearing pain, which is the predominant symptom in OA patients [2]. Clinically, immediate pain relief is obtained by a total or unicompartmental knee arthroplasty [13], in which most of the affected subchondral plate is excised. Dramatic pain attenuation is achieved with high tibial osteotomy [12], in which the high load on the abnormal subchondral bone is rebalanced. The targeting of the subchondral bone by ZOL or other bone-modifying agents, the inhibition of subchondral bone lesions and neurovascular invasion, and the improvement in subchondral bone mechanical properties would protect subchondral nociceptors from excitatory stimuli and injury and alleviate joint pain. In addition, the acidic environment that is created by increased osteoclastic activity may produce bone pain during bone destruction [47]. A significant increase in osteoclasts was observed in the abnormal subchondral bone in the MIA model, which may contribute to joint pain. An increase in subchondral bone turnover occurs with increased osteoclast activity in OA patients. Therefore, the inhibition of this increased osteoclastic activity in the subchondral bone may relieve the pain of OA.

However, the present results revealed that the inhibition of subchondral bone lesions by ZOL did not completely reverse the indexes of joint pain. One reason for this incomplete reversal may be the failure of ZOL treatment to affect the synovial reaction, which is one main source of joint pain [40]. Classical treatments that target inflammatory pain in OA [48], including acetaminophen and NSAIDs, are inadequate, and the combination of bone-modifying agents and anti-inflammatory drugs may effectively reduce OA pain.

## Conclusions

Our study demonstrated that the inhibition of subchondral bone lesions by ZOL attenuated pain behavior and reduced the expression of pain-related neurochemical indexes in the DRGs and spinal cord dorsal horns that innervated the osteoarthritic joint in a rat MIA model. The inhibition of subchondral bone lesions alleviated joint pain. These findings contribute to a better understanding of the mechanisms involved in osteoarthritic joint pain. Subchondral bone lesions should be targeted for the management of osteoarthritic joint pain.
